# Diagnostic model of saliva peptide finger print analysis of oral squamous cell carcinoma patients using weak cation exchange magnetic beads

**DOI:** 10.1042/BSR20150023

**Published:** 2015-06-22

**Authors:** Wei-Peng Jiang, Zhen Wang, Li-Xin Xu, Xin Peng, Feng Chen

**Affiliations:** * The Third Dental Center, Peking University School and Hospital of Stomatology, Beijing 100083, PR China; † Department of Oral and Maxillofacial Surgery, Peking University School and Hospital of Stomatology, Beijing 100081, PR China; ‡ Department of Central Laboratory, Peking University School and Hospital of Stomatology, Beijing 100081, PR China

**Keywords:** early diagnosis, histatin-3, matrix-assisted laser-desorption ionization–time-of-flight–mass spectrometry (MALDI-TOF-MS), oral squamous cell carcinoma (OSCC), peptides, saliva

## Abstract

Saliva diagnostics utilizing nanotechnology and molecular technologies to detect oral squamous cell carcinoma (OSCC) has become an attractive field of study. However, no specific methods have been established. To refine the diagnostic power of saliva peptide fingerprints for the early detection of OSCC, we screened the expression spectrum of salivary peptides in 40 T1 stage OSCC patients (and healthy controls) using MALDI-TOF-MS combined with magnetic beads. Fifty proteins showed significantly different expression levels in the OSCC samples (*P*<0.05). Potential biomarkers were also predicted. The novel diagnostic proteomic model with *m/z* peaks of 1285.6 Da and 1432.2 Da are of certain value for early diagnosis of OSCC.

## Abstract (precis)

Whole saliva (WS) was used for diagnosis of oral squamous cell carcinoma (OSCC); two polypeptides may be used for OSCC diagnosis.

## INTRODUCTION

Oral cancer, especially oral squamous cell carcinoma (OSCC), is a high-impact disease in the oral cavity. OSCC accounts for ~90% of malignant oral lesions and is widely recognized as the most frequently occurring malignant tumour of oral structures. Each year, approximately 500000 new cases are diagnosed worldwide, with a 5-year survival rate of only 50% [1]. In the early stages of OSCC, the tumour responds well to combination therapy, as evidenced by a 5-year survival of 80% in these patients. However, the response to treatment is much lower in advanced OSCC [2]. Thus, there is a need to investigate the molecular mechanisms involved, to identify potential therapeutic targets as well as to discover biomarkers for the early detection of OSCC and subsequent monitoring of its progression.

Saliva has gained notable attention as a diagnostic fluid because it is easy to collect and process, minimally invasive and associated with low costs [3]. It contains a large array of proteins, which may be useful for novel approaches to prognosis, clinical diagnosis and monitoring and management of disease. Comprehensive analysis of the human saliva proteome may contribute to the understanding of pathophysiologies and provide a foundation for the recognition of potential biomarkers of human disease [4–6].

MALDI-TOF-MS is a powerful technique that can be used to analyse proteins from saliva [7]. It can detect low-molecular-mass peptides with adequate resolution and sensitivity, making it a useful tool for peptide pattern profiling. In addition, beads with peptide libraries, mesoporous silica particles [8] or a magnetic core [9], such as weak cation-exchanger magnetic beads, may be utilized for selective enrichment of low-molecular-mass peptides before MS analysis. Magnetic beads constructed on nanomaterial are a promising material among the various types of separation beads. This kit based on weak cation exchange (WCX) principle. Proteins and peptides in samples are captured by specific adsorption of magnetic beads in low salt and low pH solution and released in high salt solution, so as to capture proteins and peptides in the serum. The proteins and peptides can be analysed by MALDI-TOF-MS. Using a combination of magnetic beads and MALDI-TOF MS enables efficient and sensitive detection of peptides that are specific for certain conditions; indeed, we used this technique to successfully identify serum peptide profiles in pilot studies [10,11]. This procedure, which involves weak cation-exchanging magnetic beads for sample separation, MALDI-TOF MS for peptide profile detection and a database for construction of condition-specific peptidome models, is a powerful tool that enables early detection, diagnosis and determination of the prognosis of various diseases [12].

In the present study, we investigated differences in the salivary peptide (1–10 kDa) profiles of T1 stage OSCC patients and healthy subjects by MALDI-TOF MS using a magnetic bead-based peptidome analysis of saliva samples. We aimed to identify a panel of specific biomarkers for differential expression.

## MATERIALS AND METHODS

### Ethics statement

The present study was approved by the Peking University Biomedical Ethics Committee. Adult subjects and parents of paediatric subjects signed an informed consent form before the start of research.

### Patients and saliva collection

All patients were enrolled in the Department of Oral and Maxillofacial Surgery, Peking University School and Hospital of Stomatology between April 2013 and August 2014. Samples of unstimulated whole saliva (WS) were collected from 40 OSCC patients (19 males, 21 females; aged 58.5±14.06) and 23 healthy controls for comparative analysis. The 40 OSCC patients were diagnosed according to the results of oral pathology tests and had not taken any prescription medication to prevent changes in the flow rates of saliva. Patients with other medical disorders and complicated medication requirements were excluded from this study. Detailed clinical and serological characteristics of OSCC patients are showed in Table 1. A total of 23 WS samples were collected from healthy controls with a mean age of 54.47±11.83 years. Unstimulated WS samples were collected between 9 and 10 a.m. Patients were asked to refrain from eating, drinking, smoking and conducting oral hygiene procedures for at least 1 h before saliva collection. Before collection, the subject was instructed to rinse orally with water and then rest for 5 min with his/her eyes open and head tilted slightly forward. The WS was collected over a period of 15 min or more with a paper cup on ice and then centrifuged at 2600 ***g*** for 15 min at 4°C. Then the supernatant was removed and immediately stored at −80°C in 200 μl of aliquots for further analysis. Prior to proportional peptide mass fingerprint analysis, the deep-frozen samples were quickly thawed via brief immersion into hot water to maintain the integrity of proteins.

**Table 1:** Demographics of patients (a) and healthy control subjects (b)

Patient	Diagnosis	Location	Age (years)	Sex
(a)				
1	OSCC	Right buccal mucosa	64	M
2	OSCC	Tongue	58	M
3	OSCC	Left lower gingiva	65	F
4	OSCC	Left palate	81	F
5	OSCC	Pharynx	51	M
6	OSCC	Left lower gingiva	63	M
7	OSCC	Left lower gingiva	60	M
8	OSCC	Supralabial	36	F
9	OSCC	Left floor of mouth	61	M
10	OSCC	Left upper gingiva	76	F
11	OSCC	Right lower gingiva	59	M
12	OSCC	Right mandible	65	M
13	OSCC	Left tongue	41	F
14	OSCC	Left mandible	62	F
15	OSCC	Right lower gingiva	72	F
16	OSCC	Tongue and floor of mouth	53	M
17	OSCC	Floor of mouth	46	M
18	OSCC	Ventral tongue	51	F
19	OSCC	Right floor of mouth	60	M
20	OSCC	Left buccal mucosa	68	F
21	OSCC	Left tongue	32	M
22	OSCC	Left floor of mouth	65	F
23	OSCC	Left floor of mouth	62	M
24	OSCC	Supralabial	34	F
25	OSCC	Right mandibular angle	48	M
26	OSCC	Left lower gingiva	62	F
27	OSCC	Right root of tongue	59	F
28	OSCC	Right ventral tongue	40	F
29	OSCC	Left soft palate	56	F
30	OSCC	Left ventral tongue	64	M
31	OSCC	Left tongue	70	F
32	OSCC	Right buccal mucosa	41	M
33	OSCC	Right palate	25	F
34	OSCC	Right tongue	51	F
35	OSCC	Left lower gingiva	76	F
36	OSCC	Left buccal mucosa	67	F
37	OSCC	Left tongue	21	F
38	OSCC	Right lower gingiva	51	F
39	OSCC	Right buccal mucosa	79	M
40	OSCC	Palate	55	F
Subject	Diagnosis	Age (years)	Sex	
(b)				
1	Normal	30	M	
2	Normal	31	F	
3	Normal	39	M	
4	Normal	39	M	
5	Normal	45	F	
6	Normal	50	M	
7	Normal	50	M	
8	Normal	50	F	
9	Normal	53	F	
10	Normal	53	F	
11	Normal	56	F	
12	Normal	56	F	
13	Normal	56	F	
14	Normal	60	M	
15	Normal	60	M	
16	Normal	60	M	
17	Normal	63	F	
18	Normal	67	M	
19	Normal	67	M	
20	Normal	67	M	
21	Normal	67	M	
22	Normal	67	M	
23	Normal	73	F	

### Instruments and reagents

A weak cation exchange (WCX) magnetic bead kit (Bioyong SPE-C) and robotic separation device for a 96-well plate format magnetic separator were purchased from Bioyong (Bioyong technologies Inc). An LT-2 MALDI-TOF MS (Bioyong technologies Inc) was used for the MS analysis.

### Sample application and MALDI-TOF-MS analysis

The suspension in the WCX magnetic bead kit was mixed by shaking. After eluting and more shaking, the magnetic beads were separated from the protein and the eluted peptide samples were transferred to a clean 0.5 ml of tube for further MS analysis. Then, 5 μl of hydroxy-α-cyano-cinnamic acid (HCCA) substrate solution (0.4 g/l, dissolved in acetone and ethanol) and 0.8–1.2 μl of elution were mixed and 0.8–1.2 μl of this mixture was applied to a metal target plate and dried at room temperature. Finally, the prepared sample was analysed by MALDI-TOF MS. Peptides with molecular masses of 1000–10000 Da were collected and 400 shots of laser energy were used. Peptide mass fingerprints were obtained by accumulating 50 single MS signal scans. The saliva samples collected from each patient were analysed serially three times using MALDI-TOF MS. The mean values of each sample were used for data analysis.

### Statistical analysis

The *t* test was used for comparisons between the OSCC and healthy subjects groups. Data were analysed using the BioExplorer statistical package (Bioyong Technology Inc). A *P*-value<0.05 was considered statistically significant.

## RESULTS

The general clinical characteristics of the subjects are presented in Table 1. The entire mass spectra of the extracted peptide samples from 63 subjects in the two groups were generated using the same instrument settings in the range of 1000–10000 Da (Supplementary Figure S1). Most peaks were detected in the range of 1000–3500 Da.

An average of 50 peptide mass peaks was found when the two groups were compared. Next, the peaks among the mass spectra were quantified and compared. Eight of these peptide mass peaks (1285.6, 1731, 1191.4, 1353.9, 1584.6, 1553.5, 1329.9 and 1432.2 Da) were significantly different between OSCC patients and healthy controls (Figures 1, 2 and Table 2). Among them, four peptides (at 1285.6, 1553.5, 1329.9 and 1432.2 Da) were up-regulated and four (at 1731, 1191.4, 1353.9 and 1584.6) were down-regulated in the OSCC patients (Figure 2).

**Figure 1:**
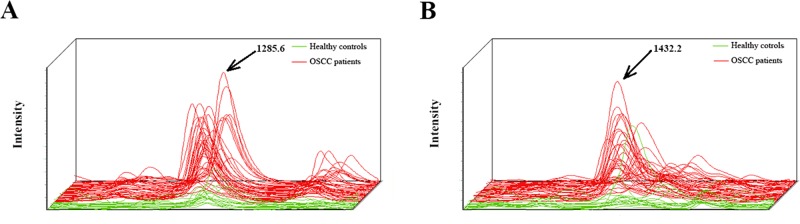
3D *m/z* ratio-intensity maps showing significantly different proteins 3D *m/z* ratio-intensity maps showed the two significantly different peptides at1285.6, 1432.2 Da, which had a particular trend among the two groups. Green curve, heathy control group; red curve, 7-month group; blue curve, OSCC patients group.

**Figure 2:**
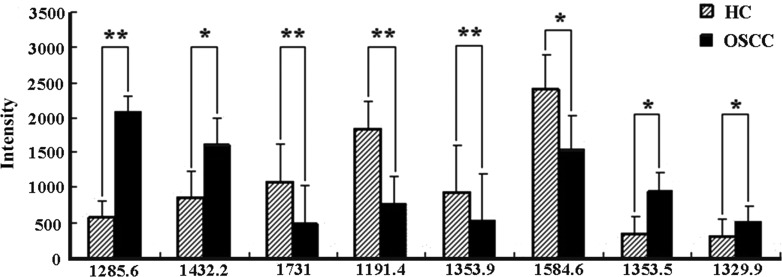
Column views of the mass spectra of the two groups The peak intensities of the two different groups showing an increasing trend in peak intensity at 1285.6, 1432.2, 1353.5 and 1329.9 Da and a decreasing trend at peak 1731, 1191.4, 1353.9, 1584.6 Da. (*P<0.05; **P<0.01).

**Table 2:** The list of peptide peak frequency greater than 50% and the *P-*value less than 0.05

M/C ratio	*P*-value	Tendency
1285.6	0.00000124	↑
1731	0.0000274	↓
1191.4	0.0000432	↓
1353.9	0.007	↓
1584.6	0.02	↓
1553.5	0.022	↑
1329.9	0.029	↑
1432.2	0.033	↑

All the eight mass peaks were used to establish the diagnostic model using the radial basis function method. Two peaks (1285.6 and 1432.2 Da) exhibited the most significant difference (*P*<0.05, by *t* test) between the two groups compared with the other combinations of peptides. Thus, we used these two peptides to establish a fitted curve. 2D-cluster plot analysis demonstrated represents the best separating peaks in 2D spaces (Figure 3), whereas 3D view of principal component analysis (PCA) scores plot analysis indicated a well differential distribution of mass peaks between controls and OSCC patients (Figure 4). Columns represent samples; rows are *m/z* peaks as indicated by the average molecular mass. The shape of the two figures showed the well-separated locations of the samples from the two groups, indicating that the fitting results were satisfactory.

**Figure 3:**
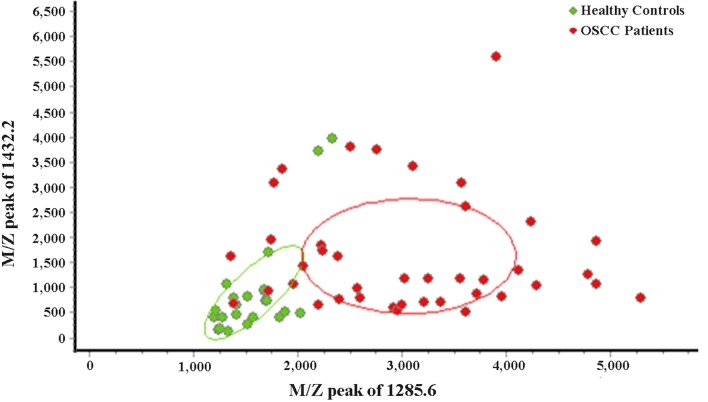
Plots of the two groups generated by combining the 1285.6 and 1432.2 Da proteins The scatter plots showed a well-fitting curve of two peaks with a significant difference (*P*<0.01, by *t* test) in their distribution between healthy controls and OSCC patients.

**Figure 4:**
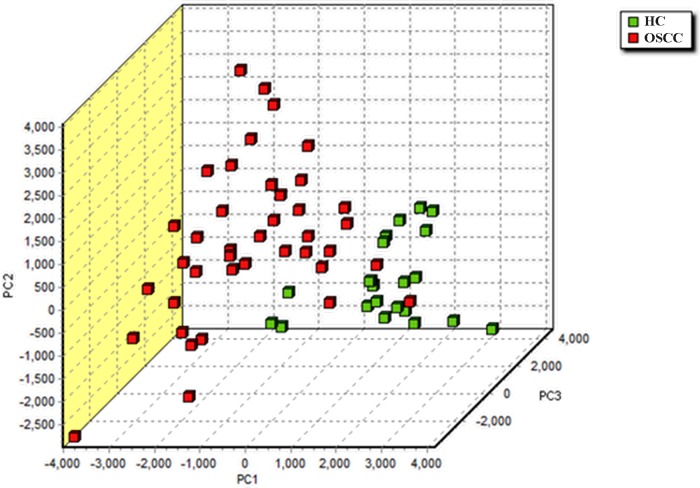
3D view created by PCA analysis 3D view displays of the principal component analysis of peptide profiles using BE software. Blue spots represent control individuals; red spots represent OSCC patients.

## DISCUSSION

Detection of oral cancer at an early stage is important for successful clinical therapy [13]. Patients with OSCC often present with advanced-stage disease, which is associated with poorer prognosis. Late-stage OSCC also requires more aggressive therapy, which results in increased functional disability. Conventional diagnostic techniques, including direct inspection and imaging technology such as positron emission tomography-computed tomography, are limited in their ability to detect early stage OSCC and are ineffective for screening high-risk populations [9]. Screening tools are needed that combine high sensitivity and specificity and are sufficiently non-invasive and inexpensive to enable widespread use.

In recent years, interest in saliva for clinical purposes as an alternative to other body fluids, such as blood and urine, has increased. WS is a complex biological fluid due to the many processes involved in its production. In addition to the exocrine components, there are several non-exocrine contributors such as desquamated epithelial cells, intact and partial blood cells, gingival fluid and possibly fluid entering the oral cavity through mucosal seepage. This renders diagnosis of disease by the analysis of saliva both challenging and attractive. Saliva was found to be similar in microbial profile to the soft tissues [14]. This was a significant finding from the study of the OSCC-free population [15]. So the screening test of salivary peptides for OSCC is appealing. MS-based proteomics is a high-throughput method used to analyse salivary proteomics and has been employed in the study of protein/peptide spectra, biological marker spectra, as well as single biological markers for complicated diseases such as cardiovascular and cerebrovascular diseases, OSCC and neuro-degenerative diseases.

To date, more than 2000 peptides have been discovered in the salivary peptidome [16–18]. By mapping the corresponding protein entries, it has been possible to assign those peptides to 695 non-redundant protein species [18]. Since the 1970s, salivary peptides have been grouped into six structurally-related major classes [19], namely, histatins, basic proline-rich proteins (bPRPs), acidic proline-rich proteins (aPRPs), glycosylated proline-rich proteins (gPRPs), statherin and cystatins [20–22]. Salivary PRPs, as well as bPRPs, aPRPs and gPRPs, are usually identified from the small peptide fraction (< 3 kDa). Some PRPs, along with statherin and histatin-1, appear to actively participate in tooth mineralization. Histatins, especially histatin-3 and histatin-5, which are found in high amounts in saliva, are strongly anti-fungal [23]. The cystatin class comprises five major isoforms (S, C, D, SA and SN), which have strong bactericidal and virucidal properties [24]. Statherin, a multifunctional molecule that possesses a high affinity for calcium phosphate minerals, such as hydroxyapatite, contributes to the maintenance of the appropriate mineral solution. Defensins are a family of low-molecular-mass (3–4 kDa) cationic proteins with antibiotic, anti-fungal and anti-viral properties. They are involved not only in innate immunity against infections but also in adaptive immunity, inflammation and wound repair [25].

In the present study, WCX magnetic beads and the MALDI-MS technique were employed to investigate WS samples from OSCC patients and healthy controls. The components extracted by the WCX magnetic method could be either low-molecular-mass peptides or fragments resulting from proteolytic activity occurring in the WS after secretion into the oral cavity. WCX magnetic beads separate the proteins and/or peptides of different isoelectric points from complex biological fluids with specific anionic ligands. The techniques of MALDI-TOF-MS combined with WCX magnetic beads incorporate both of their advantages [26]; the low cost, the simple purification, could capture more proteomes than other methods especially in the low-molecular-mass range [27]; sensitive, fast and essential for clinical use [28] allowed the identification of comprehensive ‘fingerprints’ of protein profiles within biological fluids and were used to identify biomarkers of various diseases. The effectiveness of this combination of techniques has been confirmed in many saliva-based peptide profile identification studies [29,30]. We examined 40 T1-stage OSCC saliva samples and 23 healthy control samples. Eight *m/z* peaks were found to be significantly different between the groups. Four of these were up-regulated and four were down-regulated. The mass peaks of 1285.6 and 1432.2 were detectable in all OSCC samples at a high intensity, but seldom in the healthy subjects, suggesting that these represent markers of OSCC and may play a role in the occurrence and development of this disorder. The 1731 and 1353.9 mass peaks were detected in the majority of healthy subjects, but seldom in OSCC patients so it may be others biomarkers to detect OSCC.

Our results also differ from those of Jou et al. [9]. They found three significantly different peaks in OSCC patients and healthy controls. Two of these (*m/z*=2919, 4373) were up-regulated and one (*m/z*=5592) was down-regulated. The differences between the two studies may be attributable to the different age and sex of the participants involved and/or to the different methods used to extract low-molecular-mass peptides. The 95.7% of participants enrolled in Jou et al.'s study were men (45 male, two female) with a mean± S.D. age of 50.79±10.20 years for the OSCC patients and 76.7% were men (23 male, seven female) with 44.9±10.1 years for the healthy control subjects, whereas our study used 42.5% men (17 male, 23 female) with a mean ± S.D.

Age of 56.25±14.23 years in OSCC patients and 56.5% men (13 male, 10 female) with 54.74±11.83 years in healthy controls. It is possible that the WS proteome changes with age [30]. The small peptides (<10 kDa) used for MS analysis in our study were extracted from WS samples by WCX magnetic beads, whereas the saliva samples used in Jou et al.'s study were precipitated by using C8-magnetic beads. Nevertheless, the different processing methods could lead to artificial losses and modification of the samples, which could influence the results significantly [31].

The peptide sequence identifications made in the present study have led to interesting speculations. The mass peaks of 1285.6 and 1432.2 were both identified as histatin-3 by matching these peaks to the mass spectrum database of Bioyong Technologies Inc. histatin-3 belong to the histatin family which are a class of peptides named according to their high histidine content [21] that were identified in human saliva approximately 30 years ago [32,33]. Histatin family consist of 12 members found in the saliva secreted by the salivary glands of humans and higher primates, are localized in human oral tissues [34]. Histatin-3, which is 32 residues in length, is encoded for by the histatin-3 precursor (HIS2) gene [35]. Histatin-3 could kill *Candida albicans*, the most common and the most pathogenic oral Candida species [36,37]. However, histatin-3 are also active against other yeasts and fungi, including *Candida glabrata*, *Candida krusei*, *Saccharomyces cerevisiae* and *Cryptococcus neoformans* [38,39] and some bacterial species, including Streptococcus mutans, *Porphyromonas gingivalis* and *Actinobacillus actinomycetemcomitans* [40,41].

It has been reported that microorganisms, especially Candida species, are closely associated with OSCC [42–46]. Patients with OSCC tend to possess significantly raised concentrations of certain bacteria in their saliva [15,47,48]. Previous studies by various investigators have demonstrated a significant correlation between oral candidiasis and oral squamous carcinoma in a number of studies [49,50]. Rehani et al. [51] identified Candida as a possible factor in the development of OSCC. Marttila et al. [52] found that Candida colonization frequency and density were higher at oral mucosa of OSCC patients than in healthy controls'. Oral microorganisms inevitably up-regulate cytokines and other inflammatory mediators that affect the complex metabolic pathways and may thus be involved in carcinogenesis [46]. It has been suggested that Candida species play a role in oral carcinogenesis by triggering nitrosamine compounds to activate specified oncogenes, thereby initiating oral neoplasia [51,53,54]. Previous studies were also demonstrated that the secreted anti-microbial proteins responsible for combating oral candidiasis include the salivary histatins [55,56]. So it suggests that the high level of histatin-3 in OSCC patients' saliva our findings were modulated by the raised concentrations of oral candidiasis.

In addition, histatin-3 are also involved in cell proliferation through the regulation of heat shock cognate protein 70 (HSC70) and cyclin-dependent kinase inhibitor 1B (p27Kip1) in oral cells [57] and could also bound to HSC70 inhibits HSC70-mediated activation of toll-like receptor (TLR) 4 signalling activation [58]. TLRs are a family of transmembrane proteins that recognize a variety of endogenous and microbial agents. The TLR 4 could lead to more aggressive, invasive behaviour of OSCCs [59]. It indicated that the histatin-3 may be involved in the progression of OSCC by interacting with TLR 4.

There is agreement that anti-microbial treatment is important pre-, during and post-therapy for oral cancer patients [46,60–62]. Histatin-3 possesses potent anti-fungal and anti-microbial properties and has the advantage over conventional synthetic azole or polyene anti-fungals and anti-microbial of being a naturally occurring compound in man, with no known cross-reactivity with human cells or tissues [63]. These qualities make it an ideal compound for development as an anti-fungal agent in the treatment of fungal infections of the oral cavity [64]. An important consideration in the development potential of histatin as a therapeutic agent would be the determination of the *in vivo* mechanism, occurrence- and significance of resistance to this peptide.

In conclusion, our results suggested mass peaks of 1285.6 and 1432.2 Da which were both identified as histatin-3 in saliva as correlated with OSCC progression. However, the discovered candidate biomarkers need to be extensively validated with wider cases. Clearly, it is challenging to translate candidate biomarkers from proteomic investigations into real-world diagnostic or prognostic applications. Approval of use of histatin-3 as a biomarker to detect early stage of relies on the results of large-scale multicentre clinical trials. We plan to undertake such a study in the future.

## Abbreviations

aPRP: acidic proline-rich protein
bPRP: basic proline-rich protein
gPRP: glycosylated proline-rich protein
OSCC: oral squamous cell carcinoma
TLR: toll-like receptor
WCX: weak cation exchange
WS: whole saliva
